# Coronary Microvascular Dysfunction: Bridging the Diagnosis–Treatment Divide in Women with INOCA—A Review

**DOI:** 10.3390/jcm14176054

**Published:** 2025-08-27

**Authors:** Alaukika Agarwal, Ronak Patel, Omar K. Khalique

**Affiliations:** 1 Department of Internal Medicine, Staten Island University Hospital, New York, NY 10305, USA; 2 Department of Internal Medicine, SUNY Downstate Health Sciences University, Brooklyn, NY 11203, USA; 3 Division of Cardiovascular Imaging, St. Francis Hospital and Catholic Health, Roslyn, NY 11576, USA

**Keywords:** coronary microvascular dysfunction, women’s cardiovascular health, INOCA, diagnosis–treatment gap, coronary flow reserve

## Abstract

Coronary microvascular dysfunction (CMD) is increasingly being recognized as a significant contributor of ischemic heart disease, particularly affecting women with angina and non-obstructive coronary arteries. This contemporary review synthesizes recent landmark evidence (2022–2024) revealing a striking paradox in CMD management. While diagnostic capabilities have advanced dramatically—with CMD now identified in 41% of patients with non-obstructive coronary disease—this diagnostic success has not translated into therapeutic benefits. Recent meta-analyses demonstrate that CMD doubles cardiovascular risk (HR 2.08–2.45), yet the first randomized trial of invasive endotyping (CorCTA) found that improved diagnosis failed to improve symptoms despite a 4-fold enhancement in diagnostic accuracy. This diagnosis–treatment gap represents one of the most pressing challenges in contemporary cardiovascular medicine, reflecting fundamental failures that demand urgent reconceptualization. We examine current evidence on its prevalence, diagnostic approaches, and prognostic implications, highlighting the urgent need for CMD-specific therapies to bridge the gap between diagnostic capability and clinical outcomes. Until CMD-specific therapies emerge from dedicated research programs, clinicians must optimize available treatments while advocating for the resources and research focus this condition deserves.

## 1. Introduction

Cardiac perfusion is physiologically modulated with the help of coronary microcirculation, consisting of vessels with a diameter less than 500 μm—pre-arterioles, arterioles and capillaries [1]. This microcirculation regulates coronary blood flow and fundamentally responds to increased cardiac oxygen requirements [2]. Coronary microvascular dysfunction (CMD) refers to the impairment of this mechanism. Several cardiovascular risk factors such as diabetes, metabolic syndrome, smoking, and chronic illnesses can cause endothelial dysfunction and lead to CMD [3,4,5]. Since CMD involves the microvasculature, patients with typical symptoms of angina often have normal or minimal coronary artery disease (CAD) on angiography, which leads to misdiagnosis and the dismissal of symptoms [6]. This pathophysiology has led to coronary syndromes that distinctly differ from coronary artery disease (CAD) and may be termed as Angina with Non-Obstructive Coronary Arteries (ANOCA), Ischemia with Non-Obstructive Coronary Arteries (INOCA) or Myocardial Infarction with Non-Obstructive Coronary Arteries (MINOCA) [7,8].

**Figure 1:**
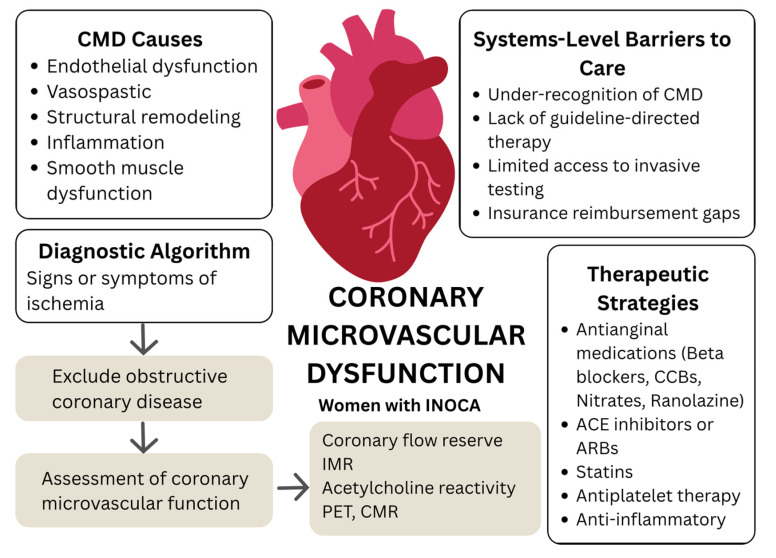
Central illustration: CMD in women with INOCA.

Currently, the following criteria have been established for the diagnosis of microvascular angina [9]:

1. The presence of symptoms suggestive of myocardial ischemia.
2. The objective documentation of myocardial ischemia as assessed by currently available techniques.
3. The absence of CAD (<50% coronary diameter reduction and/or fractional flow reserve (FFR) > 0.80).
4. Confirmation of a reduced coronary blood flow reserve and/or inducible microvascular spasm (Figure 1).

Despite this formal distinction, emerging evidence suggests CMD should be conceptualized more broadly as it appears to be a part of systemic small-vessel disease syndrome that disproportionately affects women [10]. This connects CMD to other female-predominant conditions such as heart failure with preserved ejection fraction (HFpEF), cerebral small-vessel disease, pre-eclampsia, and pulmonary hypertension via shared endothelial and inflammatory pathways [10]. Adopting this multisystem perspective with phenotyping based on clinical patterns, for example, microvascular and vasospastic angina, and physiologic metrics provides a foundational frame to guide research and care. Integrating invasive and non-invasive diagnostics into a precision medicine model by linking each physiologic endotype to targeted therapies promises to shift CMD beyond descriptive classifications [11,12].

## 2. Diagnostic Assessment of CMD

There has been a recent advancement in multimodality imaging in diagnosing and managing CMD, including cardiac magnetic resonance, positron emission tomography, and invasive functional assessment. However, despite these evolutions and increased ability to diagnose CMD, we continue to utilize previously established medications that do not improve clinical outcomes in patients. This review examines recent studies to highlight this knowledge gap and the urgent need for therapeutic innovation.

### 2.1. Non-Invasive Assessment of CMD

There are several imaging modalities for the diagnosis of CMD, including non-invasive and invasive imaging modalities. Among non-invasive imaging modalities, Positron Emission Tomography (PET) has emerged as the gold standard for diagnosis [13,14]. PET provides a quantitative assessment of myocardial blood flow (MBF) and myocardial flow reserve (MFR). MFR is the ratio of stress MBF compared to resting MBF, and a value of MFR < 2 is universally accepted as a cutoff for CMD [14].

Cardiac magnetic resonance (CMR) is another validated, non-invasive method of diagnosis CMD [13]. CMR calculates the myocardial perfusion reserve index (MPRI), which is the ratio of MBF during vasodilatory response to MBF at rest. Studies suggest an MPRI threshold between 1.7 and 1.8 to screen for CMD [13]. It has also been found that women with suspected CMD have a significantly lower mean pharmacological stress MPRI of 1.71 in contrast to asymptomatic subjects with an MPRI of 2.23 (*p* < 0.001) [15].

CT perfusion is a recent advancement that combines CT angiography with dynamic and static imaging to assess microvascular dysfunction [13]. Despite its advantage of providing both structural and functional assessment, it is lacking in robust validation for the use of diagnosis of CMD [16]. Echocardiography is another imaging modality that is common in medical practice but less robust for routine CMD assessments compared to PET or CMR [17]. A coronary flow velocity reserve (CFVR) of less than 2.0–2.5 is used to define microvascular dysfunction [18]. Single-photon emission computed tomography (SPECT) is performed after a radiotracer such as technetium−99 m or thallium−201 is injected at rest or during stress. In patients with non-obstructive CAD, SPECT may show perfusion abnormalities without the presence of significant stenosis [19], suggesting the presence of CMD. Dynamic SPECT allows for the calculation of MBF and MFR, improving the ability to diagnose CMD [20].

Emerging evidence highlights oxygenation-sensitive cardiac MRI (OS-CMR) as a recent advance in non-invasive CMD assessment. This modality utilizes the paramagnetic properties of deoxyhemoglobin, enabling the real-time imaging of myocardial oxygenation exploiting intrinsic MR signal changes induced by deoxyhemoglobin without the need for intravenous contrast or pharmacological stress agents. OS-CMR uses standardized vasoactive breathing maneuvers—a combination of hyperventilation followed by expiratory breath-hold—as controlled physiological stimuli to assess coronary vascular function [21,22]. Standardized protocols typically involve a period of free breathing, followed by paced hyperventilation around 60 s and a post-hyperventilation breath-hold around 30 s. The myocardial oxygen response during these phases is quantified as the breathing-induced myocardial oxygen reserve (B-MORE), which reflects vascular reactivity and microvascular function [21]. A 2025 case–control study showed that OS-CMR can objectively detect blunted myocardial oxygenation responses in patients with long COVID, indicating endothelial dysfunction underlying persistent cardiovascular symptoms [22].

### 2.2. Invasive Assessment of CMD

Invasive coronary angiography with adjunctive tests can directly evaluate microvascular function. There are several invasive techniques that can be used to diagnose CMD, especially when non-invasive imaging is inconclusive. These functional measures help confirm a CMD diagnosis once obstructive CAD is excluded. The following provides an assessment of invasive imaging approaches for CMD:

- Coronary Flow Reserve (CFR): Invasively measures the maximum hyperemic to basal coronary blood flow, using a Doppler flow wire (CFRdoppl) or thermodilution wire (CFRthermo) [23,24]. CFR less than or equal to 2.0–2.5 is considered suggestive of CMD [25].
- Index of Microvascular Resistance (IMR): A quantitative measure of minimal achievable microvascular resistance calculated using a pressure or temperature sensor-tipped guidewire. It calculates distal coronary pressure and the mean transit time of a saline bolus during maximal hyperemia [26]. An IMR equal to or greater than 25 suggests abnormal microvascular function [25].
- Hyperemic Microvascular Resistance (HMR): Measured with the help of a Doppler flow wire to calculate the ratio of mean distal coronary pressure to mean hyperemic flow velocity [26]. HMR greater than or equal to 1.9–2.0 mmHgcm^−1^ is above the normal threshold.
- Intracoronary Provocation Testing: Assesses microvascular spasm or hypercontractility with the help of provocative agents such as acetylcholine, ergonovine during angiography to detect microvascular or epicardial spasm [25].

These invasive assessments can identify specific CMD endotypes, such as those with impaired vasodilation, increases resistance, and microvascular spasm [27,28], and various indices are associated with worse cardiovascular outcomes [29,30].

## 3. Clinical Algorithm for CMD Diagnosis and Management in Women

The complexity of CMD diagnosis and the heterogeneity of available testing modalities necessitate a structured clinical approach, particularly for women who represent the majority of affected patients. Recent guidelines from the American Heart Association/American College of Cardiology (AHA/ACC) and the Coronary Vasomotion Disorders International Study Group (COVADIS) provide evidence-based frameworks for a systematic evaluation.

High clinical suspicion of CMD should be maintained in women presenting with specific clinical patterns. Primary indicators include typical anginal symptoms in the setting of normal or minimal coronary artery disease (<50% stenosis), particularly in perimenopausal and postmenopausal women aged 45–65 years [31,32]. The female predominance of CMD reflects biological and methodological factors, including hormonal influences on microvascular function and potential underdiagnosis in traditional diagnostic paradigms [31].

Clinical presentation patterns that warrant CMD evaluation include exertional angina lasting more than 10 min, chest pain triggered by emotional stress or cold exposure and symptoms poorly responsive to sublingual nitroglycerin [33]. Associated symptoms of dyspnea, fatigue, and palpitations often lead to frequent emergency department visits and repeated cardiac evaluations without a definitive diagnosis [32].

Cardiovascular risk factors relevant to CMD in women extend beyond traditional ones—hypertension, diabetes, and dyslipidemia—to hormonal factors such as menopause, polycystic ovary syndrome, and gestational diabetes history, along with inflammatory and metabolic conditions like autoimmune diseases and insulin resistance [34,35].

**Figure 2:**
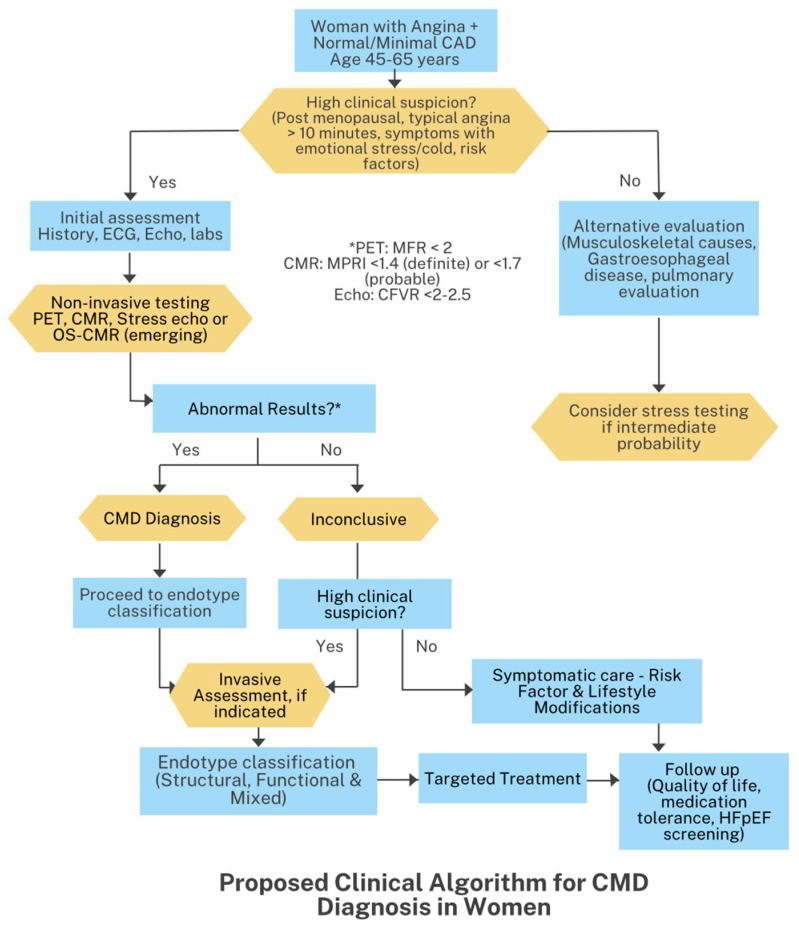
Clinical algorithm for CMD diagnosis in women. * denotes thresholds for an abnormal non-invasive result: PET MFR < 2.0; CMR MPRI ≤ 1.4 (definite) or < 1.7 (probable); stress echo CFVR < 2–2.5.

The diagnostic approach follows a hierarchical strategy prioritizing non-invasive assessments before invasive assessments. The initial assessment includes a comprehensive history and physical examination, focusing on symptom characterization and cardiovascular risk factor evaluation. Figure 2 presents a proposed clinical algorithm for evaluating women with angina or INOCA. It begins with risk stratification and non-invasive testing (stress imaging), followed by invasive coronary function testing when CMD is suspected. Early identification should triage patients so that can either receive medical therapy or undergo further evaluation (including workup for alternate causes of chest pain). The algorithm highlights that symptomatic women with persistent ischemia merit a functional assessment rather than reassurance after a “normal” angiogram.

Current treatment strategies remain largely empirical pending the development of CMD-specific therapies. Women-specific considerations require particular attention to hormonal factors, psychosocial elements and comorbidity patterns [36]. Postmenopausal women may benefit from discussions regarding cardiovascular protective hormone replacement therapy, while patients with polycystic ovary syndrome require a consideration of insulin-sensitizing therapies. The higher burden of depression and anxiety in women with CMD necessitates comprehensive stress management interventions.

## 4. CMD Endotypes and Phenotypes

Contemporary research highlights that CMD consists of distinct structural and functional endotypes, suggesting that this disease pathology requires precision medicine within this domain. This stratification would enable clinicians to move from generic treatment approaches to precise interventions that focus and treat the pathophysiology driving the disease in each patient.

### 4.1. Structural CMD Endotype

This endotype is characterized by an elevated IMR (greater than or equal to 25) and either a normal or reduced CFR [37]. This reflects fixed microvascular resistance due to capillary rarefaction, arterial hypertrophy and cardiac fibrosis [26]. The structural CMD endotype is prevalent in patients with traditional cardiovascular risk factors such as hypertension, diabetes and hyperlipidemia [26]. Subsequently, the management of this endotype involves aggressive risk factor modification and the implementation of vascular protective therapies such as statins and ACE inhibitors [38]. It is important to note that these interventions address underlying vascular remodeling and fibrosis; however, evidence for these interventions needs further studies [39].

### 4.2. Functional CMD Endotype

The functional CMD endotype is defined by reduced CFR (<2–2.5) with a normal IMR, which highlights impaired coronary vasodilation in the absence of structural microvascular abnormalities [40]. This pathology is frequently observed in younger patients without classic cardiac risk factors but the predominance of metabolic and neurohormonal disturbances [26]. It stands to reason that this endotype responds to metabolic modulators such as ranolazine or trimetazidine and functional interventions that improve vasodilatory response [26,38]. However, randomized clinical trial data for this approach are limited.

### 4.3. Mixed CMD Endotype

This endotype represents a combination of both structural and functional abnormalities resulting in severe disease with a complex pathophysiology and I associated with the poorest prognosis [26,37]. Given the mixed burden, the mixed CMD endotype requires multimodal approaches that target all contributing mechanisms [26].

While endotype classification has a clear mechanism and theoretical appeal, clinical implementation is complicated by limitations in current evidence for outcome improvement through endotype-specific therapy. Most available studies indicate symptomatic and possible quality of life benefits with tailored treatment, but long-term outcome data are lacking and reliance on expert census is common [39,41]. This requires dedicated therapeutic trials on long-term data that validate precision medicine.

Clinicians may also describe CMD using phenotypes such as microvascular angina (predominant chest pain with evidence of CMD), vasospastic angina (epicardial spasm) and mixed angina [28,29]. These phenotypes likely overlap with endotypes: for instance, pure microvascular angina often corresponds to the functional endotypes, whereas mixed angina entails combined mechanisms. Aligning phenotypes with endotypes supports a precision medicine approach: treatments can be stratified by phenotype such that vasospasm requires nitrates or calcium channel blockers (CCBs), whereas microvascular-dominant angina might benefit more from agents improving microcirculatory flow [28].

While endotyping offers insight, its implementation faces challenges. Current data suggest symptomatic benefits from tailored regimens, but long-term outcome evidence is limited. Trials of single-target drugs such as endothelin-A blockage have so far shown no clear benefit [42]. These mixed results highlight the need for well-designed trials: identifying molecular or genetic markers such as *endothelin-1* gene variants could help select patients for specific therapies. Thus, while CMD endotyping is an advancement in precision medicine, it must be validated in prospective trials.

## 5. Comorbidity Interactions

CMD and Heart Failure with Preserved Ejection Fraction (HFpEF): The relationship between CMD and HFpEF simulates microvascular–cardiac dysfunction synergy. A study demonstrated that 81% of HFpEF patients have CMD without obstructive CAD, suggesting that microvascular dysfunction is the primary driver of HFpEF [43,44].

CMD and Diabetes: Patients with diabetes experience accelerated microvascular disease through advanced glycation end products, oxidative stress and endothelial dysfunction, creating a compounding effect of CMD [45,46]. This represents a “double hit” wherein systemic and coronary-specific microvascular pathologies synergistically worsen outcomes [47].

CMD and Hypertension: Hypertensive heart disease fundamentally alters microvascular structure through arteriolar remodeling and rarefaction, creating substrate for functional CMD [48]. This demonstrates why hypertensive women display particular vulnerability to INOCA syndromes [49].

Critical insight: These comorbidity interactions challenge traditional cardiovascular risk factor paradigms. Rather than independent additive effects, CMD represents a final common pathway where multiple risk factors converge to create a clinically significant microvascular pathology requiring integrated management approaches.

## 6. Prevalence of CMD

CMD has a 50% preponderance in women [31]. However, it is well-known that women often present with “atypical” symptoms of angina and conventional non-invasive stress tests are designed to identify obstructive CAD [37]. CMD has been associated with traditional cardiovascular risk factors; however, it does not account for the significant variability in disease pathology in women [50]. This leads to an underdiagnoses of CMD in women, who are in turn undertreated, contributing to significant morbidity and adverse cardiovascular outcomes.

The systematic review conducted by Mileva et al., (2022) compiled 56 studies with 14,427 patients and found that the prevalence of CMD was approximately 41% (Table 1) [51]. Similarly, the CorCTA trial provides the detailed phenotyping of coronary vasomotor disorders [52]. Based on comprehensive invasive testing results, patients were diagnosed with microvascular angina alone, vasospastic angina alone or both conditions with a prevalence of 55%, 11.7% and 7.4%, respectively. In total, 171 of 231 patients were diagnosed with coronary vasomotor disorder, with merely 26% found to have normal coronary arteries.

Women are disproportionately affected when compared to men. The meta-analysis conducted by Mileva (2022) found that women have a 45% higher risk of CMD when compared to men (RR 1.45, with 95% CI 1.11–1.90) [51].

## 7. Outcomes of CMD

Over the last few years, several studies have emerged that further our understanding of CMD. Two comprehensive meta-analyses have quantified its prevalence and prognosis, while the CorCTA trial represents the first randomized evaluation of systematic invasive endotyping. These studies, complemented by authoritative reviews on nuclear imaging and preventive cardiology in women, provide an evidence base for examining the current state of CMD diagnosis and management (Table 2).

## 8. Diagnostic and Prognostic Indicators

The meta-analysis conducted by Jensen et al. (2023) demonstrated prognostic indicators of CMD [53]. The study compared outcomes in 4612 patients with reduced CFR compared to 11,367 with normal CFR. The outcomes were all-cause mortality and major adverse cardiovascular events (MACEs) including death, myocardial infarction, revascularization, and heart failure hospitalization. In 6 studies used in this meta-analysis, all-cause mortality was shown to have an adjusted hazard ratio of 2.45 (95% CI 1.37–3.53, *p* < 0.001) and 11 studies reported an MACE rate of 2.08 (95% CI 1.54–2.63, *p* < 0.001). This study also explores different CFR thresholds for different imaging modalities (refer to Table 3). Jensen et al. found similar adverse prognosis in both sexes when CFR is abnormally low, with three studies specifically comparing outcomes (Table 4) [53].

## 9. The CorCTA Trial

The British Heart Foundation-sponsored CorCTA trial was the first randomized clinical trial to test whether a systematic invasive assessment of CMD improves outcomes. In this trial, adding full coronary functional testing (including CFR and spasm provocation) quadrupled diagnostic yield but yielded no difference in symptoms or major events. In essence, confirming CMD did not change 6- or 12-month angina scores compared to usual care. This finding revealed a critical gap; despite knowing who has CMD, clinicians had no evidence to reduce their risk or increase their quality of life [52] (Table 5).

Despite the dramatic improvement in diagnostic accuracy demonstrated in the CorCTA trial, there was no improvement in clinical outcomes (Table 6).

## 10. Disconnect Between Diagnosis and Outcomes

CMD is a common presentation in women, especially those with cardio-metabolic risk factors [56]. While with recent advances in diagnostic imaging, both invasive and non-invasive modalities have improved the identification of CMD, the current management strategies remain empirical. Studies indicate that CMD is prevalent in 41% patients, with women facing 45% higher risk [51]. The results of the CorCTA trial show that despite a four-fold improvement in diagnostic accuracy, clinical outcomes did not show any improvement [52], revealing a critical gap between the diagnosis of CMD and treatment.

CMD involves both structural and functional dysfunction in the coronary microcirculation [25]. This impairs the ability to regulate coronary blood flow in response to increased myocardial demand through endothelial dysfunction, vascular smooth muscle cell dysfunction, structural remodeling, autonomic dysfunction and inflammation and oxidative stress [57]. This mismatch between supply and demand leads to myocardial ischemia, even in the absence of obstructive CAD. This pathophysiology has led to coronary syndromes that distinctly differ from CAD and may be termed as ANOCA, INOCA or MINOCA [7,8]. CMD and coronary vasospasms are often underlying causes involved in these syndromes [58], with a prevalence of 40–50% in women [59,60]. This makes CMD a major contributor to symptoms of angina in women.

In the Women’s Ischemia Syndrome Evaluation (WISE) study, 47% of women experienced a decrease in coronary flow reserve, which was suggestive of CMD [61]. However, it is important to note that CMD and CAD often co-exist. Studies suggest that in patients diagnosed with CAD, CMD is also present [62]. In a cohort study, 81% of heart failure with preserved ejection fraction patients were diagnosed with CMD, without obstructive CAD [43].

In the CorCTA trial described above, invasive endotyping was utilized to assess CFR and IMR and provocative testing. The diagnostic yield improved four-fold; however, there was no significant difference observed in symptom relief, quality of life or MACE [52]. This draws attention to the development of effective, targeted therapies for treatment of CMD.

## 11. Therapeutic Approaches

Current guidelines for the treatment of CMD include pharmacotherapy, risk factor modification and lifestyle interventions. Beta blockers, such as atenolol and nebivolol, are considered to be first-line therapy for symptom relief [63,64,65]. These are especially useful in patients with an increased resting heart rate or those with exercise-related angina. Nebivolol has vasodilatory properties, which is favorable on microcirculatory tone [64]. Calcium channel blockers such as amlodipine, diltiazem and nifedipine have been shown to increase exercise tolerance and are especially useful in patients with symptoms at rest or suspected vasospasm [63]. However, some patients may experience the worsening of symptoms with calcium channel blockers [63].

Angiotensin-converting enzyme inhibitors or ACE inhibitors are part of risk factor management, although direct evidence for symptom relief is limited [63]. Statins such as pravastatin have been shown to improve CFR [66]; however, rosuvastatin does not improve microvascular function [67]. Ranolazine, nicorandil, ivabradine and other medications may be considered in refractory cases [65].

Structured cardiac rehabilitation programs have been shown to increase functional capacity, reduce symptoms and enhance quality of life in patients with CMD [63].

Treatment strategies for ANOCA are severely limited and include heterogenous patient cohorts, with different endotypes [68]. Several drugs have not been approved for the treatment of ANOCA in the United States by the Food and Drug Administration (FDA) [64]. Anti-inflammatory therapies and antiplatelet therapy deserve to be studied in the context of improving clinical outcomes in patients diagnosed with CMD. There are very few randomized controlled trials of a large caliber that specifically assess treatment strategies for CMD [69,70]. Additionally, long-term treatment data are not available regarding treatment approaches and MACE and quality of life outcomes.

## 12. Emerging Therapies and Future Directions

To date, there have been few large, definitive treatment trials in CMD. One recent example is PRIZE (Precision Medicine with Zibotentan in Microvascular Angina), a randomized, placebo-controlled study of the selective endothelin-A receptor antagonist (Zibotentan) in patients with microvascular angina [42]. This study comprised 118 participants, of which 60% were women. Patients underwent exercise treadmill testing after zibotentan (10 mg dose) or a placebo and then crossed over. The PRIZE trial found no significant difference in exercise duration or ST-segment ischemia between zibotentan or placebo and also reported a high rate of drug-related side effects [42]. This “neutral” result may suggest under-dosing, the inclusion of patients without endothelin-driven disease, or the heterogeneity of microvascular pathology.

In a small pilot study, 20 women were treated for refractory microvascular angina (INOCA, CFR ≤ 2.5) by administering intracoronary autologous CD34+ endothelial progenitor cells after G-CSF mobilization [71]. At 6 months, the mean CFR rose substantially (statistically significant) and measures of angina and quality of life improved markedly, suggesting microvascular angiogenesis. However, this trial had no control group and a small sample size, putting it at a high risk of a placebo effect. It was also limited to a short follow-up. These results, nevertheless, provide a foundation for a regenerative approach that can improve CMD physiology and symptoms, encouraging future RCTs on CD34+ therapies.

The future of CMD involves large-scale molecular profiling to identify CMD subtypes and the discovery of novel biomarkers and therapeutic agents. Future trials should also test tailored therapies to specific mechanisms such as anti-inflammatories for inflammation-driven CMD, antifibrotic agents for fibrotic remodeling CMD and endothelin antagonists in genetically selected patients. Precision medicine trials using endothelial or inflammatory biomarkers for a targeted patient population may improve outcomes. Agents under study may include anti-IL-1 therapies, PDE inhibitors, or novel microvascular vasodilators.

Developments in imaging could improve CMD diagnosis and phenotyping. OS-MRI is an advancing technology in this field of research. Embedding coronary function testing into routine catheterization workflows could facilitate randomized trials that test therapies triggered by physiology. For instance, a registry-based trial could randomize patients with confirmed CMD to different treatment algorithms at the time of angiography.

Growing evidence also suggests that pre-eclampsia or breast cancer therapy could be linked to later CMD [72,73]. Studies should examine pregnancy-associated CMD with longitudinal studies of women with pre-eclampsia or gestational diabetes to assess postpartum microvascular function. Additionally, cardio-oncology CMD should be investigated in cancer survivors treated with anthracyclines or VEGF inhibitors who may develop microvascular injury.

## 13. Critical Analysis of Current Evidence

While these studies enhance our understanding of CMD, there are several limitations that must be acknowledged. In the meta-analysis conducted by Mileva et al., (2022), there is significant heterogeneity in the diagnostic criteria and patient populations across the included studies [51]. CFR thresholds also vary depending on imaging modality, which may affect prevalence estimations. The meta-analysis conducted by Jensen et al. (2023), on the other hand, demonstrates a distinct significance of reduced CFR and discusses studies with substantial heterogeneity in outcome definitions and follow-up periods [53]. The non-significance of adverse prognosis observed in males and females highlights the possibility of underdiagnosis in men or sex-specific differences in disease expression.

It is important to note that many of the therapies for CMD are borrowed from obstructive CAD care and offer inconsistent benefits in CMD. For instance, while some statins modestly enhance CFR [74,75], others have no effect, and beta-blockers can sometimes worsen microvascular spasm [76,77]. It is crucial to highlight that current treatments only provide symptomatic relief, and no medical interventions have proven to reverse microvascular pathology or reduce hard endpoints in CMD [31,70]. The CorMicA trial did show that when therapy was guided by invasive physiology, angina improved [12], illustrating some benefit from stratified care. But large-scale, CMD-specific RCTs are lacking, leaving a knowledge gap.

One consequence of this empirical approach is that CMD management often treats the patient and not the disease. Expert reviews note the paucity of randomized trials, which subsequently means we rely on heterogeneous small studies. Many antiplatelet or anti-inflammatory drugs are “deserving of study”, but their roles remain undefined. Until recently, there were no FDA-approved drugs specifically for ANOCA/CMD. Ongoing trials such as the WARRIOR trial, which evaluates the role of high-intensity medical therapy in women with INOCA, may provide further evidence [70] on risk factor treatments, but targeted drugs are urgently needed.

Beyond pathophysiology, system-level issues hinder progress. As discussed, women with CMD often do not receive the treatment they deserve in the setting of a healthcare system focused on obstructive CAD. In many hospitals, catheterization labs do not routinely perform microvascular testing, and few specialists are trained in CMD management. Insurance reimbursement may not cover newer tests or chronic therapies for CMD. These gaps mean that even when CMD is diagnosed, patients may not be guided into the invasive treatment of trials. Overcoming these barriers will require education, advocacy and changes in practical guidelines.

CMD in women represents a clear example of how medical paradigms must evolve. The assumption that a normal angiogram is translated to a benign prognosis has been proven inconclusive. CMD is common and harmful, yet our traditional workflows and therapies have not kept up with this. To bridge the diagnosis–treatment divide, we must challenge our assumptions: treat ischemia even when arteries are clear, and treat patients, not angiograms. We advocate for a research agenda and clinical strategy grounded in sex-specific precision medicine. This means routinely including women in CMD trials, analyzing outcomes by sex, and explicitly studying female-specific factors such as menopause, pregnancy as modifiers of CMD. CMD should be viewed as a systematic small-vessel disease and cardio-metabolic syndrome. And this means pursuing novel therapies guided by pathophysiology rather than empirical extrapolation.

As Munshi et al. mention, the misconception that women are “protected” from heart disease has led to under-diagnosis and undertreatment [78]. For CMD, dispelling this myth requires generating robust female-specific data and recalibrating guidelines. For example, future practice guidelines should explicitly recommend microvascular testing in symptomatic women with normal coronaries, rather than treating them as “low risk”. Healthcare systems must invest in training and pathways for CMD, as they do for heart failure or arrhythmias. If we succeed, CMD could become the foundation of precision cardiology in women. We could use endotypes and biomarkers to tailor therapies and leverage data to find responders to each drug and consequently improve outcomes. Until then, CMD remains a gap between what we can diagnose and what we can cure.

This review has several limitations that merit acknowledgement. As a narrative rather than systematic review, study selection may reflect author bias toward certain research areas or findings. While the focus on 2022–2024 highlights recent publications, this may lead to the exclusion of work published before 2022 that provides context for recent developments. The heterogeneity in diagnostic criteria and patient populations across included studies limits definitive conclusions about optimal approaches. This review focus on CMD in women, which also limits generalizability towards male patients with CMD. Finally, the rapid evolution of technology may warrant updated conclusions as more evidence emerges from ongoing research.

## 14. Conclusions

Contemporary evidence from 2022 to 2024 reveals coronary microvascular dysfunction as a prevalent, high-risk condition affecting 41% of patients with angina and non-obstructive coronary arteries, with women facing a 45% higher risk of developing this condition. While diagnostic capabilities have reached remarkable sophistication—enabling accurate identification through multiple modalities and demonstrating 4-fold improvement with systematic testing—the failure to translate diagnostic accuracy into improved clinical outcomes represents a critical challenge in cardiovascular medicine.

The diagnosis–treatment paradox exposed by the CorCTA trial demands urgent action. We can identify patients at risk and quantify their elevated cardiovascular risk (a 2.08 to 2.45-fold increased risk of death and MACE), but we lack effective treatments to alter their clinical trajectory. This gap between diagnostic excellence and therapeutic inadequacy represents one of the most pressing unmet needs in contemporary cardiology.

There are several gaps in the literature surrounding CMD, the disease pathology and, more importantly, therapeutic approaches. CMD encompasses significant heterogeneity, which limits treatment advances. This must inform future research. Novel therapies that target microvascular disease pathways must be developed. Efforts must be made to optimize diagnostic–treatment pathways, with a special emphasis on women. The increased prevalence of CMD also highlights the need for system-level implementation with training programs for CMD recognition, convenient access to CFR assessment and appropriate reimbursement.

For the millions of affected patients, particularly women who comprise the majority of CMD cases, closing this gap requires coordinated efforts in research, clinical practice, and health policy. Until CMD-specific therapies emerge from dedicated research programs, clinicians must optimize available treatments while advocating for the resources and research focus this condition deserves.

## Figures and Tables

**Table 1:** Prevalence of coronary vasomotor disorders in patients with non-obstructive CAD [51].

Condition	Prevalence (95% CI)	Detection Method
Coronary microvascular dysfunction	41% (36–47%)	Invasive: 43% (33–53%); Non-invasive: 42% (36–49%); *p* = 0.993
Epicardial vasospasm	40% (34–46%)	Acetylcholine: 49% (38–55%); Ergonovine: 48% (39–57%); *p* = 0.935
Microvascular spasm	24% (21–28%)	Acetylcholine testing
Combined CMD and vasospasm	23% (17–31%)	Comprehensive testing

**Table 2:** Summary of contemporary evidence of CMD (2022–2024).

Study	Study Type	Population	Sample Size	Key Findings
Mileva et al., (2022) [51]	Systematic Review and Meta Analysis	Patients with NOCAD	56 studies; 14,427 patients (65% women)	• CMD prevalence: 0.41 (95% CI: 0.36–0.47) • Epicardial vasospasm: 0.40 (95% CI: 0.34–0.46) • Microvascular spasm: 0.24 (95% CI: 0.21–0.28) • Women at higher risk: RR 1.45 (95% CI: 1.11–1.90)
Jensen et al., (2023) [53]	Systematic Review and Meta Analysis	Patients with NOCAD and CMD	19 studies; more than 15,000 patients	• Adjusted HR for death: 2.45 (95% CI: 1.37–3.53) *p* < 0.001 • Adjusted HR for MACE: 2.08 (95% CI: 1.54–2.63) *p* < 0.001 • Similar prognosis in both sexes
Ruddy et al., (2023) [54]	Narrative Review	Patients with NOCAD and CMD	Review	• NOCAD prevalence ~50% of angina patients • CFR measurement feasible with PET and SPECT • CFR < 2.0 predicts poor outcomes
Bullock-Palmer et al., (2023) [55]	Narrative Review	Women with CVD	Review	• Women have greater prevalence of NOCAD and CMD • CAC > 0 confers greater CV mortality risk in women (HR 1.26, 95% CI 1.08–1.48) • INOCA associated with higher MACE
Sidik et al., (2024) [52]	Randomized Controlled Trial	Outpatients with angina and NOCAD	250 screened; 231 randomized (64.5% women)	• Microvascular angina: 55.0% • Vasospastic angina: 11.7% • Both: 7.4% • Diagnostic accuracy improved 4-fold (OR 4.05, 95% CI 2.32–7.24, *p* < 0.001) • No improvement in symptoms (*p* = 0.36)

**Table 3:** Diagnostic methods and associated cardiovascular risk [53].

Method	Patients Studied	Death—OR (95% CI)	MACE—OR (95% CI)	CFR Threshold
**TTE**	5 studies	4.25 (2.94–6.15), *p* < 0.001	6.98 (2.56–19.01), *p* < 0.001	2.0–2.25
**PET**	9 studies	2.51 (1.40–4.49), *p* = 0.002	2.87 (2.16–3.81), *p* < 0.001	1.5–2.0
**Invasive**	4 studies	2.23 (1.15–4.34), *p* < 0.018	4.61 (2.51–8.48), *p* < 0.001	2.0–3.0
**CMR**	1 study	Not reported	2.62 (1.24–5.52), *p* = 0.012	1.5

**Table 4:** Sex differences in CMD.

Parameter	Women	Men	RR or HR	Source
**CMD Prevalence**	Higher risk	Reference	RR 1.45 (1.11–1.90)	[51]
**Prognosis with low CFR**	Similar	Similar	No significant difference	[53]
**CAC > 0 mortality risk**	Higher risk	Reference	HR 1.26 (1.08–1.48)	[55]

**Table 5:** Diagnostic methods comparing intervention and control group [53].

Diagnostic Stage	Intervention Group (n = 115)	Control Group (n = 116)	*p*-Value
**Pre-randomization**			
**Vasomotor disorder diagnosed**	51 (44.3%)	55 (47.4%)	0.891
**Diagnostic certainty**	18 (15.7%)	20 (17.2%)	0.943
**Post-randomization**			
**Vasomotor disorder diagnosed**	88 (76.5%)	55 (47.4%)	<0.001
**Diagnostic certainty**	102 (88.7%)	20 (17.2%)	<0.001
**OR for correct diagnosis**	4.05 (95% CI: 2.32–7.24)		<0.001

**Table 6:** Clinical outcomes of CorCTA trial [52].

Outcome	Intervention Group	Control Group	*p*-Value
Seattle Angina Questionnaire Summary Score (mean ± SD)			
**Baseline**	55.5 ± 19.9	54.1 ± 20.7	-
**6 months**	59.2 ± 24.2	60.4 ± 23.9	0.36
**12 months**	63.7 ± 23.5	66.0 ± 19.3	0.36
Treatment Satisfaction at 12 months			
**Global satisfaction**	69.9 ± 22.8	61.7 ± 26.9	0.013
Secondary Outcomes			
**Quality of life (EQ-5D-5L)**	No difference	No difference	0.992
**Cardiovascular events**	16/115 (13.9%)	11/116 (9.5%)	0.314

## Abbreviations

The following abbreviations are used in this manuscript:

CMD	Coronary Microvascular Dysfunction
CAD	Coronary Artery Disease
MACE	Major Adverse Cardiac Events
OR	Odds Ratio
PET	Positron Emission Tomography
TTE	Transthoracic Echocardiography
HR	Hazard Ratio
NOCAD	Non-Obstructive Coronary Artery Disease
INOCA	Ischemia with Non-Obstructive Coronary Arteries
MINOCA	Myocardial Infarction with Non-Obstructive Coronary Arteries
CFR	Coronary Flow Reserve
CMR	Cardiac Magnetic Resonance
